# Supplementary material for: Virulence and transmission vary between Usutu virus lineages in Culex pipiens
Source: PLoS Negl Trop Dis. 2024 Jun 27;18(6):e0012295. doi: 10.1371/journal.pntd.0012295 (PMC11236178; doi:10.1371/journal.pntd.0012295)
Supplement: S1 Table — (DOCX) [file pntd.0012295.s001.docx]

**Supporting information**

**Table S1:** **Statistical analyses of infection prevalence in mosquito 14 days post infection.**

|  | **Infection prevalence** | | |  |
| --- | --- | --- | --- | --- |
| **Variable** | χ² | d.f. | *p-value* |  |
| Lineage | 0.065 | 1 | 0.798217 |  |
| **Bloodmeal titer** | **7.61** | **1** | **0.005813** |  |
| Tissue | 0.19 | 2 | 0.908760 |  |
| Wing size | 0.0016 | 1 | 0.968380 |  |
| **Lineage:Bloodmeal titer** | **6.22** | **1** | **0.012604** |  |
| Lineage:Tissue | 0.47 | 2 | 0.791926 |  |
| Lineage:Wing size | 0.024 | 1 | 0.878020 |  |
| **Bloodmeal titer:Tissue** | **10.537** | **2** | **0.005150** |  |
| **Bloodmeal titer:Wing_size** | **5.286** | **1** | **0.021502** |  |
| Tissue:Wing size | 0.049 | 2 | 0.975920 |  |
| **Lineage:Bloodmeal titer:Wing_size** | **6.09** | **1** | **0.013587** |  |
