# Supplementary figures and images for: Virulence and transmission vary between Usutu virus lineages in Culex pipiens
Source: PLoS Negl Trop Dis. 2024 Jun 27;18(6):e0012295. doi: 10.1371/journal.pntd.0012295 (PMC11236178; doi:10.1371/journal.pntd.0012295)

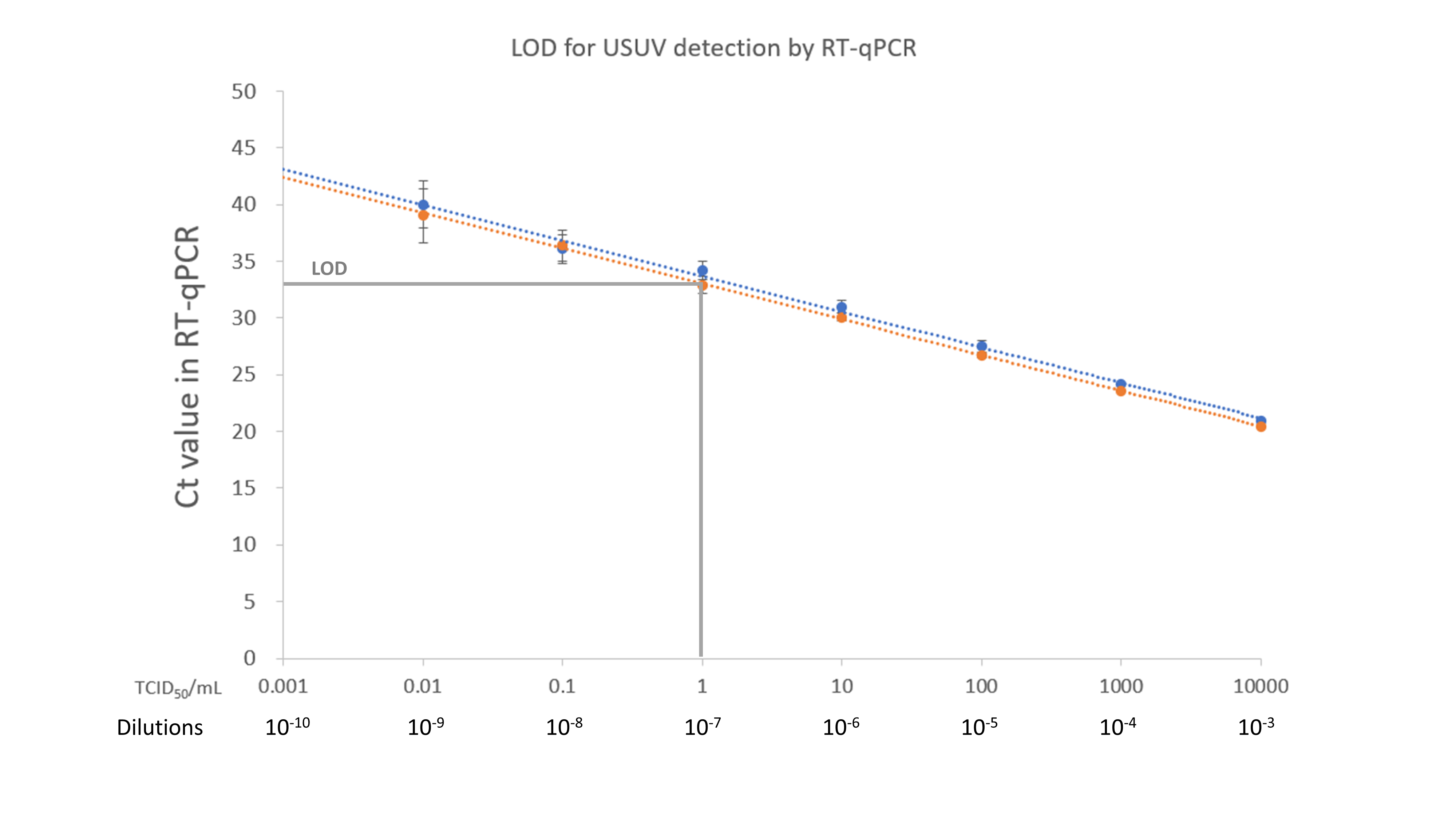

Supplement: S1 Fig — Ct values obtained by RT-qPCR (Nikolay et al. 2014 [36]) were plotted against the corresponding viral RNA titers after serial dilutions (orange for EU3 and blue for EU2). Errors bars represent the standard error of the mean. Viral stocks were titrated at 107.2 and 106.8TCID50/ml for EU2 and EU3, respectively by the method of SpearmanKärber method [34]. RNA was extracted from 150μl of virus stock using the NucleoMag RNA isolation kit (Macherey-Nagel) and eluted in 50μl before 10-fold serial dilution. RT-qPCR of serial dilutions and negative control were performed in duplicate on every plate (N = 8) containing RNA from mosquito tissues. We defined the limit of detection (i.e. individuals are considered infected or uninfected above or below this limit, resp.) as the Ct value of the last standard dilution with reproductible detection (i.e. all replicates for a dilution are positives), here 34 [95CI: 33.11; 34.96], which corresponded to the 10−7 dilution, i.e to 1 TCID50/mL or 10−3 TCID50/μL of virus stock. (TIFF) [file pntd.0012295.s002.tiff]
