# Supplementary material for: Virulence and transmission vary between Usutu virus lineages in Culex pipiens
Source: PLoS Negl Trop Dis. 2024 Jun 27;18(6):e0012295. doi: 10.1371/journal.pntd.0012295 (PMC11236178; doi:10.1371/journal.pntd.0012295)
Supplement: Attachment — Submitted filename: Response to reviewer-last.docx [file pntd.0012295.s003.docx]

**Reviewer's Responses to Questions**

**Key Review Criteria Required for Acceptance?**

**As you describe the new analyses required for acceptance, please consider the following:**

**Methods :**

**-Are the objectives of the study clearly articulated with a clear testable hypothesis stated?**

**-Is the study design appropriate to address the stated objectives?**

**-Is the population clearly described and appropriate for the hypothesis being tested?**

**-Is the sample size sufficient to ensure adequate power to address the hypothesis being tested?**

**-Were correct statistical analysis used to support conclusions?**

**-Are there concerns about ethical or regulatory requirements being met?**

Reviewer #1: The objectives of this study are clearly articulated. The authors did 3 replicates/condition (for a total of 12 independent experiments) and structured well the statistical analysis. The number of mosquitoes tested is appropriate. Ethical and regulatory requirements have been respected.

1- I have a major concern regarding the experimental set up. Why the authors choose only one time point for their analysis (14 dpi)? The choice of such long extrinsic incubation period (EIP) poses some problem: what happened before that period? Are there any difference in EIP between the strain? Considering that the authors are looking for different infectivity of the tested strains in mosquitoes, multiple time points should have been tested in order to understand the real fitness of both viral strain. A shorter EIP of one of the two strain can have a big impact in the vectorial capacity. The addition of earlier time points are also supported by the results obtained in the analysis of mortality. Considering that you are using 5-8 days old mosquitoes + 14 days of EIP = 19-22 days of survival for the vector, which under natural condition plus the additional mortality caused by USUV, is not neglectable. I suggest ,if possible, to repeat the experiment adding multiple time points (ie 3dpi (early) and 7dpi (intermediate).

We agree that analyzing multiple time points allows accurately estimating the viral dynamic within the vector, and gives a precise estimate of the extrinsic incubation period (EIP). Ideally, we would indeed have studied all the different factors influencing vectorial capacity, e.g. EIP, human biting rate, density, mosquito survival and vector competence. However, time constraints, the complexity of the experiments required (including in terms of rearing capacity in a BSL3 lab), and the necessity of replicating these experiments, led us to compromise on our goals. We thus chose to study only two among the different factors influencing vectorial capacity, i.e. vector competence and survival rate, but not EIP. We consequently set our experimental design at only one time point for analyzing these two parameters, which indeed precludes the analysis of the viral dynamics. This choice allowed us to characterize the vector competence of Culex pipiens, and, more importantly, to consider different viral titers and viral strains in the host blood (instead of only one strain and dose in most studies). Similarly, we were thus able to pursue our second objective, which was to determine whether USUV infection is harmful to mosquito (i.e. virulence), particularly in reducing mosquito survival, again considering several viral strains. To fulfill these two objectives and study the impact of infection on mosquito survival, we followed many mosquitoes individually after oral infection, the only method that enables to define the infectious status of all of them, dead or alive, throughout the 14 days: overall it represents 12 independent experiments, and ~48 females per experiment (without considering all the optimization tests required before that). It was not logistically reasonable to do more at the time, considering our staff and capacities. Finally, we have chosen 14 dpi based on published studies on the EIP of Usutu virus, West Nile virus and actually most flaviviruses (see for instance Anderson et al. 2008 10.1093/jmedent/45.3.445 for West Nile virus and Krambrich et al. 2024 10.1016/j.onehlt.2024.100707 for Usutu virus). This is the most-used time point, which allows comparing our results to most of the published data (for instance see Nikolay et al. 2012 10.4269/ajtmh.2012.11-0509; Abbo et al. 2021 10.1186/s13071-021-04686-6; Balenghien et al. 2008 10.1089/vbz.2007.0266; Vogels et al. 2016 10.1186/s13071-016-1677-0). We have added a reference line 144.

2- The authors give a general introduction about the different USUV lineages and some more details about EU2 and EU3 (Line 81-83, reference is missing). However, the authors should have include some more detail about current circulation and incidence of this two lineages in Europe. This could have justify better the choice of this two lineages, beside the different virulence.

We thank the reviewer for pointing out the lack of reference in the introduction (Cadar et al. 2022 has been added in line 86). The Usutu virus is a relatively new emerging pathogen in Europe, and most of the data on Usutu circulation is partial. We presented these limited details about the viral lineages in the discussion (line 346-347) from the publication of Cadar et al. 2022 10.3390/v15010077. To our knowledge no more recent data has been published, despite surveys performed before 2020, and partial information on recent circulation have been obtained from personal communications with operators in charge of the national West Nile virus surveillance. Based on data from several European countries and years, EU2 was the most prevalent lineage in several host in southern and central Europe and EU3 was the most found lineage in northern Europe (Vilibic-Cavlek et al. 2020 10.3390/pathogens9090699). The differential impact of two of these viral lineages on a mammalian model (Cf. study by Clé et al. 2021 10.1186/s12974-020-02060-4) is one of the major starting points of our study, and mainly drove our choice. In the revised manuscript, we provided more details in the introduction (line 86-88).

3- The authors used RT-qPCR to quantify the virus. This approach is overall good but careful should be used. Normally other techniques, such as plaque assay or focus forming assay, should be used to assess vector competence in order to really calculate the number of infectious viral particle. Detection of viral genome is not synonyms of infectious viral particles. In the case of TE (saliva sample) where the viral titer is generally low this difference is even more important. If possible i suggest to test at least positive samples by PFA or similar.

We agree with the reviewer comment that in our study, we only detected and quantified viral genomes using the RT-qPCR, but not infectious viral particles. This approach has the advantage of quantifying viral replication whether or not each replication is associated with the formation of an infectious viral particle. Thus we used the RT-qPCR results as an estimation of replication within mosquito, and as a proxy for transmission. Other suggested techniques can no longer be used, as our samples were fully used for RNA extraction. We clarified this in discussion section (line 1128-1130 of the revised mansucript)

4- The survival data are very interesting, but the data are not well explained. I suggest to rewrite the description, clearly stating what type of sample (live/dead mosquitoes, which day have been excluded etc etc) was used in each graph. The figure quality should be improved. The colored area is not clear and overlapping colors make it difficult to understand. The legend should be improved as well (color code, area meaning).

Thank you for pointing this lack of clarity in the figure captions. Figures 4 and 5 were detailed in the result section only (from line 284 to 330 of the original manuscript); more info are now summarized in the captions of the revised manuscript (See last versions of figures). Note that the figure colors are color-blind-friendly, and we used standard representation of the 95% confidence intervals.

Reviewer #2: See summary

Reviewer #3: (No Response)

**Results :**

**-Does the analysis presented match the analysis plan?**

**-Are the results clearly and completely presented?**

**-Are the figures (Tables, Images) of sufficient quality for clarity?**

Reviewer #1:

1- The authors claim to have tested vectorial capacity multiple times throughout the manuscript. However, by analyzing Infection, dissemination, and transmission, they assessed vector competence. This should be modified throughout the manuscript.

We agree with the reviewer’s comment. Infection, dissemination, and transmission allow to directly analyze vector competence, and not vectorial capacity. However as stated in line 89 of the submitted manuscript vectorial capacity is a multifactorial trait, and we provided detailed explanation of its equation. By studying mosquito survival and particularly the survival of infected mosquitoes, we could also analyze vector/pathogen interactions: for instance whether virus infection impacts life history traits. We indeed focused on vector competence and survival only (see also answer to Method comments), but, with the assumption that all other factors are equal, it also allows to estimate the relative vectorial capacity, cautiously. To avoid misunderstanding, we made changes in line 142, 271, 1081, 1098, 1277 and 1285 of the revised mansucript.

2- In lines 466-468, the authors calculate the vectorial capacity. However, it is not clear how they did it. No information about vector density and daily host biting ratio is present in the manuscript, nor have they explained how they calculated/estimated the other parameters in Table 6 (or i don't understand it). This aspect should be clearly reflected in the Materials and Methods and Results sections. Personally, I don’t think the authors should try to calculate vectorial capacity but they should focused only vector competence and the effect of the two tested strain in the mortality.

We have added a section in the material and methods (line 263-268) to explain in details how the relative vectorial capacity has been calculated (see previous comment), as suggested by the reviewer, to clarify our results. We however kept the discussion of the revised manuscript about these estimates of vectorial capacity (again all other things being equal), because it is key to understand how variations of both vector competence and survival (the latter being the most influential parameter) can each affect vectorial capacity and transmission.

3- The authors frequently use tendency in the description of the results (ie line 225-229). But in my opinion, if a result is not statistically significant speaking about tendency means nothing (this is why we use statistical analysis). i suggest to not use tendency to avoid confusion. Limit the results to what is really significant.

The p-value (an estimate of the statistical test significance) indicates the likelihood that the observed data support the null hypothesis (here H_0_ = the prevalences are equal): a p-value >0.05 thus only indicates a lack of evidence for rejecting the null hypothesis H_0_, but it is not equivalent to providing evidence for H_0_ (i.e. that there actually is no difference between groups in our data; Chen et al. 202310.1016/j.patter.2023.100878). Without estimates of type II error, a p-value >0.05 might thus only mean that the data do not provide evidence strong enough to reject: there could still be a real difference, but it might be smaller or more variable than our experimental design was able to detect H_0_ (i.e. the experiment might lack power). In this respect, we think that discussing the tendencies, particularly when there are consistently in the same direction over several replicates/experiments, is a more conservative and cautious approach. Despite our efforts, the overall number of replicates remains indeed limited for high statistical power.

4- Tables are well designed but i suggest to move Table 4 (nothing is statistically significant) in supplementary material (Table 3 and table 4).

In our study, we aimed at determining the virulence of each USUV lineages on mosquito. We measured mosquito survival after blood-feeding, comparing the effect of infectious blood versus non-infectious control, and comparing the infected mosquitoes with the non-infected but exposed mosquitoes. Testing the effect of viral load on survival is key to determine whether virulence is associated with the number of pathogens, as expected under the virulence-transmission trade-off hypothesis (Alizon et al. 2008 10.1016/j.tpb.2008.04.003). Therefore table 4 presents a central result, even if no statistically significant effects were observed.

5- I find the discussion to long, mainly because of systematic repetition of results. I suggest to delete unnecessary repetition or move it to appropriate chapter: ie: introduction (Line 345-349) or Results (Line 400-403; 431-436).

We have removed some unnecessary sentences (line 1101, 1171 and 1213 of the revised manscript)

6- Line 361: The experiment was done under controlled environmental conditions, so how can this be a differential factor?

The mosquito colony was reared in insectary, all experiments were performed in the BSL3 laboratory, and mosquitoes were held in climatic chambers. While several parameters such as temperature, hygrometry, feeding resources are controlled, there still could be some variations which cannot be avoided. Indeed, we provided the temperature and hygrometry with a range value. However, the design of our experiment assures that the different replicates and treatments have experimented the same conditions, so that comparison is possible and so that differences between them should not result from distinct environmental conditions.

7- Line 400-403: Although there is a high titer in the thorax (and midgut), this does not translate into a greater dissemination capacity, suggesting the absence of a dose-dependent mechanism. Do the authors have any insights into this phenomenon

Viral infection in mosquito thorax indicates dissemination from the midgut to the rest of the mosquito body. To determine the ability of the virus to disseminate, we measured the proportion of mosquito with infection in the thorax among those with an infected midgut (dissemination rate). Independently of whether a virus can disseminate, variations of replication can be observed. Thus a higher viral load in the thorax does not mean a greater dissemination. We observed that a higher viral load in midgut did not lead to a higher proportion of mosquito with disseminated infection for both lineages and at both titers (Figure 2). However, the observed transmission rate may indicate that, for EU3 lineage only (see Figures 2 and 3), a higher viral load in the thorax could increase the likelihood of virus infection in the saliva. There may be a dose-dependent transmission efficiency for EU3 but not for EU2 lineage. This pattern is consistent with a higher replication rate of EU2 or with a lineage specific salivary gland barrier. This has been added in the revised manuscript (line 1169-1171).

8-Line 404-406: This finding contrasts with your dissemination data, where no differences were observed regardless of blood titer usage or viral titer reached in the midgut. Indeed, the midgut escape barrier appears to be unaffected by viral titer but rather depends on the virus's physical ability to cross the basal lamina (see reference 10.1038/s41564-019-0619-y)

We agree with the reviewer comment, our dissemination data showed no differences between lineages or between viral titers, suggesting undifferentiated midgut escape barrier (i.e. USUV lineages could reach other tissues outside the midgut similarly). However, these data do not contrast with our statement in line 416 that suggested the USUV lineages differed in replication, not dissemination. Replication could be defined as the number of viral particles produced in one tissue, whereas dissemination is the propagation of virus to pass from one tissue to another. We precised that in the revised manuscript (line 1173 of the revised manuscript).

9-Line 412: analysis of early and intermediate EIP should be added to verify this hypothesis

We agree with the reviewer that it would have been nice to measure EIP too (see also method answer), and indeed suggested in the original manuscript to investigate early and intermediate time points in order to test our assumption (line 1182-1196 and 1244-1246 of the revised manuscript).

Reviewer #2: See summary

Reviewer #3: Results

1. Not sure if I understand the following statistics and statements. “IE (midguts), only the “Lineage” variable had a significant effect with a lower prevalence for EU2 than EU3 (χ² = 4.29; p-value = 0.03, Tab. 1 and Fig. 1A) (low titer: IEEU2 = 0.31 ± 0.19 and IEEU3 = 0.44 ± 0.22, p-value > 0.05; high titer: IEEU2 = 0.44 ± 0.21 and IEEU3 = 0.61 ± 0.21, p-value > 0.05; Fig. 1A”

Figure 1A does not show any significant difference in prevalence for either lineage.

The statistical analysis indicated that the variable lineage is significant but the lineage x blood meal titer interaction appeared not significant. We thought that providing a figure with the mean prevalence merging both viral titers could impair clarity. Consequently, Figure 1 showed the prevalence of infection for each lineage and blood meal titer separately, thus showing their interaction.

2. Overall, figures 1 and 2 are part of the same data. The authors can merge these figures. The midgut barriers are well known for a lot of mosquito-borne viruses. This is not new information.

Figure 2 have been made from the same data as Figure 1 but showed a different facet of arbovirus-vector interaction. Figure 2 allowed to clarify the relative role of various barriers to the final infection of mosquito saliva. These barriers are indeed recognized in the literature as key in arbovirus-vector interaction, so that it seems important for us to visualize them. By comparing the rates in Fig 2, rather than the efficiencies as in Fig. 1, it is easier to observe that the midgut barrier (MIB) is not the differential barrier for the two virus lineages; conversely, the salivary gland barrier is relatively more efficient in blocking the progression of the infection, with a lineage-specific difference. The two figures are thus complementary viewpoints on the same data and provide knowledge on the two distinct features of the arbovirus-vector interaction that are analyzed, presented and discussed separately.

3. How was the prevalence calculated ? Is it a ct value on qpcr ? What’s the cut off ? The data does not make a lot of sense. e.g. low titers for EU3 lineage show around 50% mean midgut prevalence but figure 3 shows all the viral loads below zero. The same applies to thorax and saliva.

Prevalence is calculated by dividing the number of positive samples for each tissue over the total number of samples. RT-qPCR have been also performed using serial dilutions of USUV ARN extracted from viral production as standard and a negative control. We clarified that in supplementary data with the figure below and in the material and method section (line 183-186). The limit of detection has been defined as the Ct value of the last standard dilution with reproductible detection (i.e. all replicates for a dilution are positives). In our study, a 10^-7^ dilution of ARN extracted from viral stocks (titrated at 10^7.2^ and 10^6.8^TCID50/ml for EU2 and EU3 respectively) was the lowest reliably detectable quantity, and it gave a Ct value of 34 [95CI: 33.11; 34.96]. 10^-8^ and 10^-9^dilution did not give reliable detection between duplicates within one RT-qPCR run or between several runs, negative control never resulted in specific amplification of Usutu. The 10^-7^ dilution corresponded to 1 TCID_50_/mL or 10^-3^ TCID_50_/µL of virus stock. We thus chose this value as detection limit: below the tissue is considered infected, above it is considered not infected. All viral loads are thus ≥0 (10^-3^ genome copies per µl is equivalent to 1 genome per ml).

**Figure S1. Limit of detection (LOD) of USUV genome by RT-qPCR.** *Ct values obtained by RT-qPCR (Nikolay et al. 2014 [36]) were plotted against the corresponding viral RNA titers after serial dilutions (orange for EU3 and blue for EU2). Errors bars represent the standard error of the mean. Viral stocks were titrated at 10^7.2^ and 10^6.8^TCID_50_/ml for EU2 and EU3, respectively by the method of SpearmanKärber method [34]. RNA was extracted from 150µl of virus stock using the NucleoMag RNA isolation kit (Macherey-Nagel®) and eluted in 50µl before 10-fold serial dilution. RT-qPCR of serial dilutions and negative control were performed in duplicate on every plate (N=8) containing RNA from mosquito tissues. We defined the limit of detection (i.e. individuals are considered infected or uninfected above or below this limit, resp.) as the Ct value of the last standard dilution with reproductible detection (i.e. all replicates for a dilution are positives), here 34 [95CI: 33.11; 34.96], which corresponded to the 10^-7^ dilution, i.e to 1 TCID_50_/mL or 10^-3^ TCID_50_/µL of virus stock.*

4. The comparisons in the graphs are not clear. What is the question here ? Is it to compare different lineages or high or low titers ? Just because there is significant difference between something doesn’t mean it has to be shown in the figure.

The goal of this study was to compare the vector competence for different USUV lineages, their impact on mosquito survival, and to test whether the virus titer could impact these parameters. The graphs allow to simultaneously highlight the lineage differences and the differences between titers and potentially their interactions. The significant differences are indicated.

5. What is the scale on Y axis in Figure 3 ? How do you have 10-4 genome copies per microliter ? A lot of discussion and focus is on this result. I am not sure if the scale makes sense. The limit of detection of qpcr is never discussed. Anything below 1 genome copy should be considered negative. All the stats need to be redone after removing values below 1 genome copy. I believe this will change all the results and discussion.

As explained above (answer to reviewer 3's comment n°3), the limit of detection has been defined by the Ct value of last standard dilution with reproductible detection (i.e. all replicates for a dilution are positives). In our study, a 10^-7^ dilution of ARN extracted from viral stocks was the lowest reliably detectable quantity and gave a Ct value of 34 [95CI: 33.11; 34.96]. We have added detailed information of the determination of the limit of detection in the material and method section and figure in supplementary data (line 183-186 and see above). Our limit of detection would correspond to 1 TCID_50_/mL or 10^-3^ TCID_50_/µL of virus stock. None of the values reported here are below 0 (none are negative), but can be below 1 genome/µl (e.g. 10^-7^, the chosen limit, corresponds to 1 genome/mL).

6. I am not sure if I understand the two methods of survival curve. Was every mosquito tested for infection status ? Even the dead ones every day ? How accurate is virus detection in dead mosquitoes ?

Figure 4 represent the survival curves of all mosquitoes after feeding with infectious and control blood. None of the mosquitoes reported in this figure were tested for virus infection. This allowed to test whether a simple exposition to the virus through a blood meal could impact on survival. In Figure 5, each mosquito, either dead or alive, was tested for presence of USUV infection, to test whether replicating virus in mosquitoes impacted their survival. In this case, mosquito that died 24h after blood feeding were removed because they did not digest their blood meal and virus may be detected from the undigested blood in the midgut rather than from replicating virus in mosquito tissue. Other than that detection on dead mosquito is as accurate as on live ones, as long as they are not collected more than 24h after their death. Here, dead mosquitoes were removed daily and conserved at -80°C, and were not dissected. Virus detection was performed on whole mosquitoes. We added a paragraph in the material and method section to specifically detail the survival experiments (line 200-204 and figure captions of the revised manuscript).

7. Also, the survival curves are a bit misleading. The axis starts at 70% giving an impression of huge difference in survival. Even though it is statistically significant, not sure if 80% vs 100% survival has biological implications. If there are there are, it is not discussed very well.

Y axis of survival curves have been truncated to focus on the relative survival differences. For sake of clarity and better observation, we indeed zoomed in the graphs. Figures have been modified on axis to make the reader fully aware of this zoom.

Survival rate is the most influential factor of the vectorial capacity as demonstrated by Garret-Jones in 1969 https://www.ncbi.nlm.nih.gov/pmc/articles/PMC2556109/. Indeed, vectorial capacity depends directly on the $\frac{p^{n}}{-\ln p}$ , where p is the daily survival rate and n the extrinsic incubation period (see equation 1 in the manuscript). A sensitivity analysis shows that a reduction of 10% of p (and keeping all other factors equal) will lead to a 84% reduction in vectorial capacity if the extrinsic incubation period (EIP) is 14 days (the reduction is 71% if the EIP is 8 days). Thus even a slight variation of survival can have a great impact on transmission. This was not clearly stated, so as suggested by the reviewer, it is now indicated in the discussion (line 1233-1235 of the revised manuscript).

We precisely tried to estimate the biological implications of the observed, by calculating the relative EU3/EU2 vectorial capacity using our estimates of survival and vector competence and keeping all other factors equal. We can thus discuss EU2 and EU3 transmission between mosquito and host taking into account the whole cycle, line 1269 of the revised manuscript.

8. Why did author use only one time point. Is it possible that the virus can eventually reach the same titers. Only one time point does not provide sufficient information about all the different parameters authors have calculated. Multiple time points in relevant tissues will provide a much clearer picture of viral dynamics within the vector.

We agree that estimating the infection parameters at several time points would lead to a better characterization of the viral dynamics within the vector, but as stated above (see answer to reviewer 1's comment n°1 in method), this was not the primary focus of the present study.

**Conclusions :**

**-Are the conclusions supported by the data presented?**

**-Are the limitations of analysis clearly described?**

**-Do the authors discuss how these data can be helpful to advance our understanding of the topic under study?**

**-Is public health relevance addressed?**

Reviewer #1:

1- The conclusions support the data presented and the authors discussed. However the article lack of a limitation chapter in which for example the authors can discuss the lack of early and intermediate EIP in their analysis. I am skeptical about the vectorial capacity calculation (see comment above).

*Please see detailed answers to reviewer 1's comments n°1 in method and n°2 in results. We hope that the revised version of the manuscript (particularly lines 263-268 and 1198) will alleviate these concerns.*

Reviewer #2: see summary

Reviewer #3: Discussion

1. It seems like the authors are not sure about their own data. In the first section about vector competence, authors highlight the need to generate reliable data.

In the first section of the discussion, we discussed how our data show extended variation between individuals and between blood-feeding assays. This makes us point out the necessity of replicating the experimental infection to generate reliable data, taking into account these variations, which is too often not the case in the literature, where most studies report only one replicate for each blood-feeding assay (i.e. infection). Therefore, we opened the discussion on the biases that can exist in many published studies of vector competence that lack experimental replication. There was a misunderstanding here and we modified this part to prevent it (lines 1083_1087 of the revised manuscript ).

2. It is obvious that the higher virus titer produced better infection in mosquitoes. The rationale behind using these two titers is not clearly discussed. What does it represent biologically ?

We discussed in line 352-354 of the original manuscript the influence that viral titer in host blood may have on infection and virulence presenting published data on Usutu, West Nile and St Louis encephalitis viruses. Also Clé et al. (2021) showed that EU2 replicated ~100 times better than EU3 in mouse model. Therefore, we tested how a 100-fold variation of viral titer, which represent different host viremia for EU2 and EU3, influence vector competence and influence the impact of infection on mosquito survival. We provided evidence that low virus titer still produces better infection for the EU2 lineage only contrary to what might be expected based on the hypothesis of a replicative fitness trade-off between taxonomically distant hosts (Coffey et al. 2008 10.1073/pnas.0712130105). It thus suggests that EU2 lineage might better replicate than EU3 in mosquito as well as in mouse model. We clarified the rationale of testing the two viral titers in line 147-148 of the revised manuscript.

3. Line 383, if it’s not statistically significant it shouldn’t be discussed.

We disagree with this comment. As explained above (answer to reviewer 1's comment n°3 in results), hypothesis testing implies that non-significant results cannot be ruled out, because p-value > 0.05 only indicates that there is no evidence to reject prevalence equality, i.e. inequality cannot be ruled out, it may just be an experimental power problem, particularly when tendencies are consistent over assays. We also think that negative results are worthy of being discussed.

4. Not sure If I understand the following statement “Differences between blood meal titers were not significant but prevalence increased slightly at high blood meal titer”

Again, non-significant differences do not mean absence of differences or equality. Based on three replicates, the prevalence was slightly higher at high titer, and EU3 was more prevalent than EU2 at both titers, but there are large variations between replicates, so that these differences in prevalence were not statistically significant. In the revised manuscript, we included a statistical analysis on prevalence from all tissues together, thus increasing the sample size. It showed a significant effect of “bloodmeal titer” (χ² = 7.61; p-value = 0.0058) and of the “bloodmeal titer x lineage” interaction (χ² = 6.22; p-value = 0.012) with a higher prevalence when mosquitoes fed on blood meal containing a higher virus titer than the one fed with lower virus titer for EU3 but not EU2. Infection prevalence was also significantly influenced by the “bloodmeal titer x tissu” interaction (χ² = 10.54; p-value = 0.0051) suggesting a different impact of bloodmeal titer in the different tissues. This analysis has been added in the revised manuscript (line 279-290 of the revised manuscript).

**Editorial and Data Presentation Modifications?**

**Use this section for editorial suggestions as well as relatively minor modifications of existing data that would enhance clarity. If the only modifications needed are minor and/or editorial, you may wish to recommend “Minor Revision” or “Accept”.**

Reviewer #1: (No Response)

Reviewer #2: - Mosquitoes’ death has been monitored daily for 14 days and dead mosquitoes have been subject to virus detection by RT-qPCR. These data are included in the survival study. However, it would be interesting to look at a possible correlation between the viral load and the time of death in these early dead mosquitoes. This could feed the final model where EU2 lineage has a low virulence while EU3 has a high virulence in mosquitoes and strongly affect their survival.

*We agree with the reviewer, and indeed investigated the correlation of viral load on survival using the USUV infected mosquitoes only (Line 328of the original manuscript). We performed a cox proportional hazard regression and found no correlation in our data (Table 4) probably because of low sample size. This is stated line 1088* of the revised manuscript*.*

- The results observed with the viral load (Fig 3) in the different tissues where at low titres EU3 load in the midguts and thoraces is quite low compared to EU2 and at high titres is the same level than EU2 don’t support a model (Fig 6) where EU3 lineage would replicate as much as EU2. Indeed, the authors state in the discussion (line 404) that “these observations suggest that EU2 replicates more efficiently in mosquitoes than EU3”. The model in Fig 6 should be modified (virus +++ > Virus + for EU3 in mosquitoes).

*Thank you for pointing this typo error, we have made the change.*

- In Fig 3 it seems that for EU2 high titers n=29 midguts while n=30 for thoraces. Authors should have recovered the same number of samples for these 2 conditions at max.

*Among all tested tissues, the quantification failed in one midgut and was not included in the analysis (specified as NA). This is precised now line 254-255* of the revised manuscript *.*

- Include the sample size in the appropriate figures.

*Sample sizes were added in the figure and detailed in the corresponding caption.*

- Duplicated ref 3 – ref 20

*We have made the change.*

- p20 l439: mere / more

*We have made the following change: “simple exposure to USUV EU3 virus is not enough to reduce survival” (line 1189 of the revised manuscript)*

Reviewer #3: Abstract

1. Shorten the first sentence of the abstract or break into two different sentences.

*We have made the change.*

2. What is USUV ? Describe the abbreviation once before using it.

*We have made the change.*

3. What does the following sentence imply ? Infection rate was variable among blood feeding assays but was similar between EU2 and EU3 lineages.

We have discussed the variability of the infection between individuals within one experimental group or between blood feeding assays, but the differences (or absence of difference) between the two viral lineages were consistent among replicates. This has been clarified in the revised manuscript (line 22-23 of the revised manuscript).

4. There seems to be a lot of confusion in the abstract. Please simplify the statements. In the abstract authors say “ EU2 viral load was high at all titers which suggest a greater replication”. Then in the next line “EU3 was more virulent to mosquitoes than EU2”. Is this referring to mosquito mortality ?

These two sentences indeed present two different results from our experiments. Replication and virulence are two distinct viral traits that are often expected to be linked in the literature. Virulence is the pathogen ability to cause damage to a host, and in our study it thus refers to the mortality induced to mosquitoes. As pointed by the reviewer, we have added “induced mortality” in line 31 to avoid confusion and reformulated the abstract of the revised manuscript.

5. When authors mention transmission is it bird-bird transmission ? Are humans considered the dead-end host?

Transmission refers to the mosquito-to-host transmission whatever the type of host. To date, humans are considered dead-end host as for West Nile virus (Bowen et al. 2007 10.1097/QCO.0b013e32816b5cad). This now clearly indicated line 80 of the revised manuscript.

Introduction

1. Line 67. Too long. Divide into two sentences. Line 67 reads like authors are talking about human outbreaks but the following line mentions outbreaks in birds.

This sentence is a bit long because we cited all countries where USUV has emerged. We removed all the cited countries to shorten the sentence as suggested by the reviewer.

2. Line 77 reference ?

A reference has been added

3. Line 83. Significant difference ?

Line 83 to 84: It was written significant variation: “viral pathogenesis in mice and human cell culture revealed significant among-lineage variation in virulence”. We thus refer to the significant variation. We rephrased the sentence to improve clarity (line 88 of the revised manuscript).

4. In a multi-host transmission setting what is the daily host biting rate ? Is it for birds or for humans ?

The biting rate is the combination of the gonotrophic cycle (time between two blood meals) and the relative preference of the vector to one host over all possible hosts that the vector may encounter. When estimating the transmission between two hosts (for instance from bird to human), biting rates of the two hosts are considered in the vectorial capacity equation. We have modified the equation 1 to generalize the vectorial capacity calculation for multi-host transmission.

$C=\frac{ma_{1}a_{2}p^{n}c'}{-\ln p}$ a_1_ is the biting rate on the first host (acquiring pathogens) and a_2_ is the biting rate on the second host (transmitting pathogens)

5. Did you study vector competence or transmission of the virus ? Please make sure the right terms are used.

We have studied the vector competence as well as the survival of infected mosquitoes. These two parameters allowed us to estimate the relative vectorial capacity of EU3 relative to EU2, which is defined as the potential of transmission of a pathogen by a vector population. We modified the sentence in line 136 as suggested by the reviewer.

--------------------

**Summary and General Comments :**

**Use this section to provide overall comments, discuss strengths/weaknesses of the study, novelty, significance, general execution and scholarship. You may also include additional comments for the author, including concerns about dual publication, research ethics, or publication ethics. If requesting major revision, please articulate the new experiments that are needed.**

Reviewer #1: Additional comment to the Authors:

I suggest condensing lines 87-102. This lengthy paragraph merely provides a general overview of several aspect of vector biology, which is likely familiar to the reader. Instead, I recommend focusing on providing more detailed information on other aspects more relevant to your study, such as elaborating on EU2 and EU3 (including their circulation, detection in vector and host, and outbreaks), as well as explaining the rationale for their inclusion in your research.

Thank you for this suggestion, we amended the introduction accordingly, lines 86-87 and 147-148 *of the revised manuscript*.

Minor suggestion:

Line 62: add ; after family

*Done.*

Line 63: do not capitalize virus

We have made the change line 69.

Line 71: characterized instead of typified

Done, line 74

Line 77: mutation also facilitate adaptation to vector and/or host

We have added “with potentially new hosts and new vectors” in line 80 of the revised manuscript.

Line 134: if possible add GPS coordinate

We provided the link to the website of La Tour du Valat. GPS coordinates have been added 43.509297, 4.668958.

Line 216: vector competence not vectorial capacity (see my comment above)

We have changed the sentence to be more specific (line 71 of the revised manuscript 5)

Line 394: infection and escape barrier.

We may not have understood this comment. We have stated that the escape barriers could halt or slow down viral dissemination within the mosquito vector, and infection cannot play such a role.

Line 398: add B --> Fig.2B

*done*

Line 400: add B --> Fig.3B

*done*

Reviewer #2: In this paper Prat et al, investigate the virulence of 2 Usutu virus European lineages and their ability to be transmitted by a French Culex pipiens pipiens mosquito strain. Authors assess the prevalence of USUV EU2 and EU3 in mosquitoes by measuring the infection, the dissemination and the transmission efficiencies upon oral infection with low or high viral titers.

Finally, analysis of the survival upon EU2 and EU3 infection reveals a strong negative effect of EU3 lineage on survival while EU2 significantly affect survival only at high titers. The proposed model, presented in the last figure, suggests that:

- EU2 has a low virulence in mosquitoes and a high transmission rate from mosquito to the vertebrate host in which a high virulence and a decreased survival has been previously reported

- EU3 has a high virulence in the mosquito and a high transmission rate from the Vertebrate host to the mosquito

This study is of particular interest from an epidemiological point of view, considering the recent Usutu outbreaks reported in several European countries. It also tackles the poorly studied question of the different vectorial capacity of mosquitoes relative to virus lineages. Moreover, the paper is well written, the experimental procedures detailed, the data clearly presented and the statistical analysis relevant and well explained.

*We thank the reviewer for this general comment.*

Reviewer #3: Overall, figures 1 and 2 represent a single experiment with a single time point which does not provide information about transmission dynamics. It is possible that different virus lineage may require different transmission time. Analysis with multiple time points is needed to support the model.

The data used to calculate prevalence is not stated clearly. Without the limit of detection most of the data from figure 1 and statistical analysis will change.

*As mentioned earlier (answer to reviewer 1's comment n°1 method), we did not investigate in this study the temporal dynamic of infection within mosquitoes. We analyzed how mosquito respond to infection between two virus lineages and at two distinct titers focusing on mosquito survival and vector competence. This has been clarified in the material and method section, line 213-215 and supplemental data. We also added in the same section some details on how we set the limit of detection (see answer to reviewer 3's comment n°3, results).*

Figure 3 is very confusing and does not make sense to me. My understanding is authors decided to use any mosquito with a detectable ct value as positive and used it for analysis. Although statistically you can plot a graph and get ct values with high dilutions in your standard curve, biologically it does not make sense.

*See answer to reviewer 3's comment n°5, results. We have added some details in the material and method section, line 213-215 and supplemental data.*

Figure 4 and 5, essentially show the same data. Not sure if information in figure 5 adds more value to survival curves.

*As mentioned in answer to reviewer 3's comment n°6 results, Figure 4 and 5 don't give the same information, and the comparison between the two figures provided important insights in how virulence of EU3 may be mediated in mosquitoes.*

****Prat et al. examined how differences in Usutu virus (USUV) lineages and the infecting dose affect the vector competence of locally acquired Culex pipens mosquitoes. A major finding from this study is the opposite virulence pattern in vertebrate hosts and the vectors: despite higher infection/dissemination/transmission efficiency, EU3 had lower vectoral capacity in *Cx. pipens* due to low mosquito survival compared to EU2. This result suggests lineage-specific dynamics in the virus transmission cycle: EU2 faces a barrier at vertebrate to vector transmission due to the shortened vertebrate host lifespan while EU3 overcome the same challenge at the vector to vertebrate transmission stage. Although the idea of balancing replication and virulence for optimal transmission is not novel, the idea of the lineage-specific balance is quite interesting concept and surely adds to the field.

The major concern is the limit of detection of qRT-PCR and the authors’ interpretation of the data. Infection was quantified using qRT-PCR, and specimens with extremely low viral load (10^1 GE/ul and lower) were considered positive in downstream analysis. This is a real stretch because RNA loads less than about 10 or so genome copies would not be considered actually positive by most researchers in the field, particularly in the absence of corroborating data or information on the limits of detection and/or linear range of the assay. The limit of detection by qRT-PCR should be determined by using noninfected mosquito samples and provided as supplement together with the standard curve, and LOD and/or linear ranges provided in the text and indicated on the figures. Ideally, infection should be identified by confirming the presence of infectious virus by plaque assay or observing cytopathic effect on cells. If using qRT-PCR, it is good practice to identify and use the threshold genome copy numbers at which the infectious virus is detectable through above method.

*See answer to reviewer 3's comment n°3, results for a detailed response with a figure. R*T-qPCR have been performed on serial dilutions of USUV ARN extracted from viral production as standard and on a negative control. The limit of detection has been defined as the Ct value of the last standard dilution with reproductible detection (i.e. all replicates for a dilution are positives). We *have added some details in the material and method section, line 213-215* and clarified that with supplementary data (see the figure above).

Secondly, the vectoral capacities displayed on table 6 needs more explanation. For instance, extrinsic incubation period (EIP) is a part of formula but was not studied in this research. If authors assumed EIPs of two lineages to be the same, it should be addressed.

*We clarified in the manuscript that estimation of the EU3/EU2 relative vectorial capacity in based on the two parameters measured, vector competence and survival, with all other parameters being equal. This has been clarified in the revised manuscript line 263-268. See also answers to reviewer 1's comment n°1 method and n°1 results.*

Other minor suggestions are as follows:

Reference is needed for line 74-74 “However, infections in these non-avian hosts are likely not transmissive (dead-end hosts).”

We have added the following reference (Bowen et al. 2007 10.1097/QCO.0b013e32816b5cad)

Line 91 introduces formula of vectoral capacity and line 99-101 introduce “vector competence,” which is one parameter in the vectoral capacity. It is easier to follow if vector competence is explained before the vectoral capacity.

We structured our introduction to develop current knowledge on Usutu from the virus and its epidemiology to its interactions with mosquitoes. Therefore after developing on the epidemiological and transmission cycle, we focused specifically on transmission by vectors through the vectorial capacity, and then on its key parameters.

Line 213 “Hazards Ratio” should be explained mathematically.

The hazard ratio compares the risk of occurrence of an event (here death on individuals) between two groups based on the survival curve. This has been clarified in line 262.

Fig2b caption should describe what the three asterisk means, rather than one asterisk.

Done

Line 489-490 discusses how the shortened lifespan of vertebrate hosts decreases EU2 fitness. However, EU2 does replicate to higher titer in mouse model by ~2log difference (doi:10.1186/s12974-020-02060-4), likely increasing the infectivity of engorged mosquitoes. This effect should not be ignored in discussion.

*In line 489-490, we discussed how shorten host lifespan would reduce the probability of transmission to mosquitoes and thus the overall fitness of the virus. Our experimental design indeed investigated two distinct titers in the host blood differing by 100 folds. We showed that prevalence of infection in mosquito midgut were not significantly different between EU2 at high titer and EU3 at low titer, which suggests that the transmission potential to mosquitoes of the two lineages might not be different. We have added some clarification in the discussion (line 1298-1301 of the revised manuscript).*
