# Supplementary material for: Virulence and transmission vary between Usutu virus lineages in Culex pipiens
Source: PLoS Negl Trop Dis. 2024 Jun 27;18(6):e0012295. doi: 10.1371/journal.pntd.0012295 (PMC11236178; doi:10.1371/journal.pntd.0012295)
Supplement: Attachment — Submitted filename: response to reviewer_phase2.docx [file pntd.0012295.s004.docx]

Reviewer's Responses to Questions

Key Review Criteria Required for Acceptance? As you describe the new analyses required for acceptance, please consider the following:

**Methods**

**-Are the objectives of the study clearly articulated with a clear testable hypothesis stated? -Is the study design appropriate to address the stated objectives? -Is the population clearly described and appropriate for the hypothesis being tested? -Is the sample size sufficient to ensure adequate power to address the hypothesis being tested? -Were correct statistical analysis used to support conclusions? -Are there concerns about ethical or regulatory requirements being met?**

Reviewer #1: -If the author performed the experiments in climatic chamber under controlled environmental conditions (which I understand have not been changed), those cannot be consider variable. Please remove it from the text e.i. line 395 of discussion and adress the discussion.

We have removed the environmental conditions to avoid any confusion (lines 376 – 378), as suggested.

Reviewer #2: OK

Reviewer #3: (No Response)

--------------------

**Results**

**-Does the analysis presented match the analysis plan? -Are the results clearly and completely presented? -Are the figures (Tables, Images) of sufficient quality for clarity?**

Reviewer #1: -While your understanding of p-values and the interpretation of statistical significance is correct, discussing tendencies without statistical significance can be misleading and can generate snowball effect mistake when citing (we often see a tendency became significant when cited). Scientific reporting should prioritize clarity and accuracy. Therefore, I recommend that you refrain from discussing tendencies unless they are supported by statistically significant results. This approach will ensure that the conclusions drawn from your study are based on reliable and reproducible data. e.g. remove lines 415-417.

We have removed the term tendency to avoid any confusion, and changed the sentence accordingly (lines: 26, 38, 397-399 and 499)

-While the challenges of conducting experiments in a BSL3 laboratory are acknowledged, the inclusion of multiple time points is crucial for a comprehensive understanding of viral dynamics and EIP. The current setup precludes a full analysis of these factors. I appreciate the suggestion of the Author to test early time in the discussion (line 512-513) however the manuscript should include a detailed discussion of this limitation.

We agree that our setup precludes a full analysis of the factors influencing vector competence. However, as acknowledged by the reviewer, this was not the goal of our study, as it was clearly stated in the revised manuscript. We indeed included a discussion of the limitations of our experimental design and the advantages of investigating several other factors (lines 364-372,377-381, 447-454 and 499-500).

Reviewer #2: OK

Reviewer #3: (No Response)

--------------------

**Conclusions**

**-Are the conclusions supported by the data presented? -Are the limitations of analysis clearly described? -Do the authors discuss how these data can be helpful to advance our understanding of the topic under study? -Is public health relevance addressed?**

Reviewer #1: Now that relative vectorial capacity has been used by Authors and the assumption at the base of their calculation are fully discussed i am more confident with their results.

The study has several limitations (many of them addressed in the reviewed version). I suggest to add a limitation chapter where discuss all of them in a ordinated manner.

We thank the reviewer for this comment. We have discussed the limitations (viral lineage, time points, etc..) in the corresponding paragraphs (see previous answer).

Reviewer #2: OK

Reviewer #3: (No Response)

--------------------

**Editorial and Data Presentation Modifications? Use this section for editorial suggestions as well as relatively minor modifications of existing data that would enhance clarity. If the only modifications needed are minor and/or editorial, you may wish to recommend “Minor Revision” or “Accept”.**

Reviewer #1: (No Response)

Reviewer #2: None

Reviewer #3: (No Response)

--------------------

**Summary and General Comments Use this section to provide overall comments, discuss strengths/weaknesses of the study, novelty, significance, general execution and scholarship. You may also include additional comments for the author, including concerns about dual publication, research ethics, or publication ethics. If requesting major revision, please articulate the new experiments that are needed.**

Reviewer #1: -The distinction between vector competence and vectorial capacity is crucial and should be meticulously maintained throughout the manuscript. Clear differentiation will prevent any potential misinterpretations by readers. I appreciate the effort of the Authors to clarify this matter in the manuscript and to use a relative vectorial capacity, highlight the assumption of the survival rate and biting rate.

We thank the reviewer for this comment.

Reviewer #2: The authors well took into account my suggested modifications.

Reviewer #3: (No Response)

--------------------
